# Supplementary material for: Global land and carbon consequences of mass timber products
Source: Nat Commun. 2025 May 26;16:4864. doi: 10.1038/s41467-025-60245-y (PMC12106697; doi:10.1038/s41467-025-60245-y)
Supplement: Supplementary file 1 — Supplementary Information [file 41467_2025_60245_MOESM1_ESM.pdf]

Supplementary Information

Global Land and Carbon Consequences of Mass Timber Products

Kai Lan[1,2,+], Alice Favero[3,+], Yuan Yao[1,*], Robert O. Mendelsohn[4,*], Hannah Szu-Han Wang[1]

[1]Center for Industrial Ecology, Yale School of the Environment, Yale University, 380 Edwards Street, New Haven, CT, 06511, USA
[2]Department of Forest Biomaterials, College of Natural Resources, North Carolina State University, 2820 Faucette Drive, Raleigh, NC, 27695, USA
[3]RTI International, Research Triangle Park, NC 27709, USA
[4]Yale School of the Environment, Yale University, 195 Prospect Street, New Haven, CT, 06511, USA
+These authors contributed equally.

*Co-corresponding author: y.yao@yale.edu; robert.mendelsohn@yale.edu;

Pages: 30

Table of Contents

Supplementary Note 1. Lumber production.. 3
Supplementary Note 2. Cross-laminated timber and potential benefits of substituting traditional materials.. 4
Supplementary Note 3. Landfill of wood waste 6
Supplementary Note 4. Greenhouse gas emissions of traditional wood products......................... 7
Supplementary Note 5. Projections of cross-laminated timber demand....................................... 8
Supplementary Table 1. Wood supply (Mm^3) of sawtimber for traditional wood products, pulpwood, and sawtimber for cross-laminated timber... 12
Supplementary Table 2. Forest area in the baseline scenario (Mha). 13
Supplementary Table 3. Change in global carbon stock and greenhouse gas emissions relative to the baseline between 2020-2100 if recovering 100% cross-laminated timber panels for power generation.. 14
Supplementary Table 4. Change in global carbon stock and greenhouse gas emissions relative to the baseline between 2020-2100 if recycling 50% cross-laminated timber panels for the second use and landfilling 50% cross-laminated timber panels. 15
Supplementary Table 5. Summary of cumulative greenhouse gas consequences of each cross-laminated timber scenario in 2100 compared to the baseline (no cross-laminated timber).......... 16
Supplementary Table 6. Notations and corresponding descriptions for the main text. 17
Supplementary Table 7. Ecoinvent 3.9 cut-off database processes used.................................... 19
Supplementary Table 8. Parameters for wood, lumber, and cross-laminated timber production. 20
Supplementary Table 9. Parameters for landfill of wood waste.. 21
Supplementary Table 10. Average structural material usage for 1 m^2 floor area....................... 22
Supplementary Table 11. Parameters and values for modeling greenhouse gas emissions of traditional wood products. 23
Supplementary Table 12. λ for each region floor area ridge regression model. 24
Supplementary Fig. 1. Projected demand of sawtimber for cross-laminated timber production from 2020 to 2100.. 25
Supplementary Fig. 2. Sensitivity analysis of global cumulative change in greenhouse gas emissions relative to the baseline from 2020 to 2100 in Scenario 3.. 26
Supplementary References.. 27

Supplementary Note 1. Lumber production.

The yield of bark, wet lumber, slabs/chips, and wet sawdust from sawtimber was estimated based on the literature and recorded in Supplementary Table 8 (ref.[1,2]). As mentioned in the main text, mill residues (bark, wet sawdust, dry planning, shavings/chips and sawdust) are combusted to provide the energy for the dry kiln. If not sufficient, natural gas is used; when energy is excessive, then mill residues are used for power generation[1]. The energy demand for kiln drying was determined as the total heat demand in drying divided by the overall energy efficiency for energy generation and drying. The total heat demand was determined based on the work by Bowyer et al.[3], where the desorption heat was presented in MJ energy needed for 1 kg of water to be evaporated out of wood. In this study, the slabs/chips from sawing are assumed to be combusted for power generation; the potential substitution benefits of replacing the grid electricity are included.

Supplementary Note 2. Cross-laminated timber and potential benefits of substituting traditional materials.

At the cross-laminated timber (CLT) plant, lumber preparation conducts the visual grading and grouping process. The lumber preparation groups the lumber for different layers and directions (longitudinal layers for visual grade No. 2 and transverse layers for visual grade No. 3)[4]. In this study, the lumber moisture has reached the target moisture content, so re-drying is not needed. Then end-jointing, layering, gluing, pressing, planing, and end-cutting (Computerized Numerical Control) are conducted in sequence[1,5–9]. The waste produced in the CLT plant is sent to the landfill site (see Supplementary Note 3).

In this study, the potential benefits of substituting CLT buildings for traditional reinforced concrete and steel buildings are also considered[10]. The structural material consumption for CLT buildings and steel & concrete buildings are estimated on 1 m^2 floor area basis, based on the whole building life cycle assessment (LCA) study by D'Amico et al.[10], as shown in Supplementary Table 10 (ref.[10,11]). For the upstream production of the building materials, this study uses the globally average processes from the Ecoinvent 3.9 cut-off database (see Supplementary Table 10) to determine the GHG emission reduction per m^2 floor area. The recycling rate for steel is assumed to be 65% based on the literature[11]. The rest of the steel is landfilled. The upstream production burdens of steel and concrete products and landfill of steel are derived from Ecoinvent 3.9 cut-off database (see Supplementary Table 7)[12].

Two additional conceptual end-of-life cases of CLT panels are conducted: 1) material recycling case with 50% recycling and 50% landfilling; 2) energy recovery case with 100% CLT panels combusted for power generation. For the first additional conceptual case, due to the lack of data, this study assumes 50% of recycling rate with the rest for landfilling[1,13]. The CLT panels are

assumed to be recycled back to be shaped into CLT products[1]. The greenhouse gas (GHG) emissions and energy consumption of CLT recycling are assumed to be 50% of the normal producing process[1]. Future research could update these inventory data if the inventory data of recycling CLT emerge in the future decades. For the second conceptual case, the CLT panels are combusted to generate electricity to replace the market grid electricity. The GHG emissions of the potentially replaced electricity adopts the global average value from the Ecoinvent 3.9 cut-off database (see Supplementary Table 7)[12].

Supplementary Note 3. Landfill of wood waste

Landfilling wood waste slowly releases majorly CO_2 and CH_4 (ref.[14]). To assess the GHG emissions from landfilling wood waste, there are two steps included in this study. First, CH_4-rich GHG emissions from landfill decay are estimated based on the Intergovernmental Panel on Climate Change (IPCC) First Order Decay method[1,14]. Second, since the GWP factor of CH_4 in landfill gas is high (GWP-100 27.0 for non-fossil CH_4) and has potential energy recovery value, landfill gas recovery for energy generation is assumed to be recovered and combusted for power generation[15]. All the parameters and values are collected from the literature and recorded in Supplementary Table 9.

$$C_{decomposed} = W \times DOC \times DOC_f \times (1 - e^{-k_{LF}t}) \quad (1)$$

$$CH_{4\,generated} = [(C_{decomposed} \times MCF \times F \times 16/12) \times (1 - R)] \cdot (1 - OX) \quad (2)$$

Supplementary equations (1) and (2) show the method of the IPCC First Order Decay[14]. $C_{decomposed}$ is the accumulative decomposed carbon mass to year t; W is the mass of deposited wood waste (*wet basis*); DOC the degradable organic carbon of wood waste; DOC_f is the faction of DOC that can decompose; k_{LF} is the landfill decay rate[16]. In supplementary equation (2), MCF is the methane correction factor determined by the site management[14]; F is the volume fraction of methane in landfill gas before recovery; R is the total recovered CH_4 by energy recover device[16]; OX is the average oxidation factor describing the fraction of oxidized methane[16]. This study assumes the common recovery value of R to be 0.75 based on the review work by Anshassi et al.[17]. The recovered landfill gas is combusted to generate power. The generated power is calculated based on the total lower heating value of landfill gas and electricity generation efficiency[17]. The detailed values of the parameters are shown in Supplementary Table 9.

Supplementary Note 4. Greenhouse gas emissions of traditional wood products.

To calculate carbon stock changes in traditional wood products compared to the baseline in in year t and region i ($\Delta TWP\_C_{i,t}$), three components (i.e., HL^l, f_m, $\Delta Timber\_Prod\_C_{i,t}^l$) are necessary (see equations (13–15) in the main text). $\Delta Timber\_Prod\_C_{i,t}^l$ is given by GTM as mentioned in the main text. The *HL* for sawtimber is 30 years which is averaged according to the global production share of lumber, plywood, fiber board, and particle board based on the study by Johnston et al.[18] and their half-life spans (35 years for lumber, 25 years for plywood, fiber board, and particle board[18,19]). The *HL* for pulpwood is 2 years for pulp and paper products[18,19]. f_m^l is the biogenic carbon stored in wood products after manufacturing. Similar to *HL*, the f_m for sawtimber are averaged based on the f_m of lumber, plywood, fiber board, and particle board and their corresponding global production share[18]. The f_m for pulpwood are averaged based on the f_m of mechanical pulp and chemical pulp and their corresponding global production share[18]. f_m for each wood product is collected from the literature and database[12]. The detailed values of f_m and the global production share are shown in Supplementary Table 11. To derive ${Net\_GHG_{TWP}}_{i,t}^l$ shown in equation (17) in the main text, the average emission factor EF^l is determined in the same way as f_m. *EF* for each product is also shown in Supplementary Table 11.

Supplementary Note 5. Projections of cross-laminated timber demand.

The projections of CLT demand start from projecting the total urban commercial and residential (including apartments and high-rise, excluding detached and semi-detached) building floor areas ($TotalA_{commercial\ i,t}$ and $TotalA_{residential\ i,t}$). The total urban commercial and residential building floor areas are assumed to depend on the urban population and Gross Domestic Production (GDP) per capita. The historical population and urbanization rate (urban population divided by total population) for each country are derived from the World Bank database[20,21]. The processed historical population data in 16 regions are shown in Supplementary Data 1. The historical urban residential and commercial building floor areas from 1970 to 2010 are derived from the study by Deetman et al.[22]. Note that the urban residential buildings for $TotalA_{residential\ i,t}$ only include apartments and high-rise buildings and exclude detached and semi-detached buildings that are not supposed to be replaced by mass timber products[22]. The urban commercial buildings include office, retail, hotel, and government buildings[22]. Detailed data are shown in Supplementary Data 1. Then the historical GDP per capita, urbanization rate, and historical urban residential and commercial building floor area per capita are fed into the regression model to generate the total urban commercial and residential building floor area per capita from 2020 to 2100. Note that the baseline of this study excludes current CLT supply in 2020 due to data uncertainty and its minimal contribution to the total global wood supply (less than 0.3%) based on the literature estimation[10,23].

Floor area projection was carried out with ridge regression to ensure generalizability. Every GTM region possesses two models, one for residential floor area per capita projection ($Model_{res}$), and the other for commercial floor area per capita projection ($Model_{com}$). There are 32 models in

total. The feature matrix consists of $log_{10}(gdp\ per\ capita)$, $urbanization\ rate$, and an interaction term. Before model construction, a series of data preprocessing was implemented:

(1) GDP data interpolation. The originally collected GDP data was recorded every five years from 1980 – 2008. To obtain annual GDP, we interpolated the data with a piecewise cubic polynomial, which was implemented with CubicSpline function from scipy, a python package.

(2) Filtered data for specific regions. Many regions before 1990 have low data quality due to political instability, e.g., EEU, AFME, RUSSIA, SSAF, RSAM (see Supplementary Data 1 for the region definitions in the Global Timber Model (GTM)). Thus, for those regions, only data between 1990 – 2009 was used for training.

(3) Computed GDP per capita and urbanization rate. $gdp\ per\ capita = \frac{gdp}{population}$; $urbanization\ rate = \frac{urban\ popultion}{population}$. Further, log transformation was implemented because GDP per capita was right-skewed for all regions. That is, $log_{10}(gdp\ per\ capita)$ was used in the feature matrix.

(4) Prepared the feature matrix (**X**) for model construction as shown in supplementary equation (3). Particularly,

$$\mathbf{X} = \begin{bmatrix} | & | & | \\ \mathbf{x_1} & \mathbf{x_2} & \mathbf{x_1 x_2} \\ | & | & | \end{bmatrix} \quad (3)$$

where $\mathbf{x_1} = log_{10}(gdp\ per\ capita)$; $\mathbf{x_2} = urbanization\ rate$, consists of feature data from all years (from 1980–2100).

Then, **X** was standardized. The standardized **X** was used for model construction and divided into three parts – historic data from 1980–2009 was used for model training;

historic data from 2010 was used to examine out-of-sample test error; the rest of the SSP scenario feature data (2010–2100) was used for projection.

The projection was then carried out with ridge regression to ensure generalizability. The magnitude of the regularizer λ (see Supplementary Table 12) was chosen such that they resulted in a low out-of-sample test error, respectively, for each region model. In particular, every model was trained on historic data (1980–2009), tested on the hold-out historic sample (2010), and projected for SSP scenarios (2010–2100). Covariates and outcomes are listed below. Both models have the standardized $\mathbf{X}$ as the feature matrix; $\mathbf{y_1}$ was the dependent variable for $Model_{res}$, and $\mathbf{y_2}$ for $Model_{com}$ as shown in supplementary equations (4)–(6):

$$\mathbf{y} = \boldsymbol{a}\mathbf{X} + \lambda||\mathbf{X}||_2^2 \tag{4}$$

$$\mathbf{y} = \mathbf{y_1} = residential\ floor\ area\ (for\ Model_{res}) \tag{5}$$

$$\mathbf{y} = \mathbf{y_2} = commercial\ floor\ area\ (for\ Model_{com}) \tag{6}$$

Last but not least, residential floor area and commercial floor area per capita were calculated using the projected $\mathbf{y}$ in in supplementary equations (7) and (8).

$$residential\ floor\ area\ per\ capita\ =\ \mathbf{y_1}/urban\ population \tag{7}$$

$$commercial\ floor\ area\ per\ capita\ =\ \mathbf{y_2}/urban\ population \tag{8}$$

After $TotalA_{residential\ i,t}$ and $TotalA_{commercial\ i,t}$ are generated, $A_{i,t}$ can be determined by in supplementary equation (9) as shown below. $D_{residential\ i,t}$ and $D_{commercial\ i,t}$ is the demolition rate of urban residential and commercial buildings in year *t* and region *i*, and is derived from the study by Deetman et al.[22] (see detailed values in Supplementary Data 1). $S_{residential\ i,t}$ and $S_{commercial\ i,t}$ represents the portion of urban residential and commercial buildings that adopts steel and concrete structures in year *t* and region *i*, respectively. The country-level information is

derived from PAGER that is a global building inventory database containing the data of building type in urban and rural areas in each country or region[24] (see detailed values in Supplementary Data 1).

$$NewA_{i,t} = (TotalA_{residential\ i,t} - TotalA_{residential\ i,t-1} + TotalA_{residential\ i,t} \times D_{residential\ i,t}) \times S_{residential\ i,t} + (TotalA_{commercial\ i,t} - TotalA_{commercial\ i,t-1} + TotalA_{commercial\ i,t} \times D_{commercial\ i,t}) \times S_{commercial\ i,t} \quad (9)$$

In equation (9) in the main text, the CLT usage factor (m^3 CLT per m^2 building area), f_{CLT}, is used to calculate the CLT usage based on the literate data (see Supplementary Table 10)[10]. The conversion factor for CLT from sawtimber (m^3 CLT per m^3 wet sawtimber), c_{CLT}, is derived from the process model developed for lumber and CLT production.

For the adoption rate of CLT r_t, this study assumes it follows the logistic model as one of the technology diffusion models, as shown in supplementary equation (10)[25,26]. The parameter values and adoption rate are shown in Supplementary Data 1.

$$r_t = M \frac{e^{(a+b(t-2020))}}{1+e^{(a+b(t-2020))}} \quad (10)$$

Supplementary Table 1. Wood supply (Mm^3) of sawtimber for traditional wood products, pulpwood, and sawtimber for cross-laminated timber.

Sawtimber for traditional wood products	2020	2030	2040	2050	2060	2070	2080	2090	2100
Baseline	1512	1715	1924	2052	2140	2188	2316	2342	2358
Scenario 1	1490	1695	1834	1917	1997	2067	2226	2291	2374
Scenario 2	1508	1713	1912	2003	2033	2086	2240	2301	2373
Scenario 3	1474	1663	1743	1794	1860	1927	2064	2132	2195
Pulpwood	2020	2030	2040	2050	2060	2070	2080	2090	2100
Baseline	610	781	830	921	931	1065	1140	1254	1269
Scenario 1	595	765	790	873	878	1005	1074	1195	1219
Scenario 2	607	777	820	901	893	1015	1084	1207	1259
Scenario 3	580	744	760	828	832	947	1039	1127	1185
Sawtimber for cross-laminated timber	2020	2030	2040	2050	2060	2070	2080	2090	2100
Baseline	0	0	0	0	0	0	0	0	0
Scenario 1	0	11	206	244	257	270	285	303	321
Scenario 2	0	1	9	75	208	257	276	293	309
Scenario 3	0	22	413	486	514	535	569	600	634
Total wood supply	2020	2030	2040	2050	2060	2070	2080	2090	2100
Baseline	2122	2497	2754	2973	3071	3254	3456	3595	3627
Scenario 1	2086	2471	2829	3034	3131	3341	3585	3788	3913
Scenario 2	2114	2492	2741	2980	3134	3358	3600	3801	3941
Scenario 3	2055	2430	2916	3107	3206	3409	3672	3859	4014

Supplementary Table 2. Forest area in the baseline scenario (Mha).

Year	Plantations	Natural Forest	Managed Forest	Total
2020	91	2864	944	3899
2030	106	2772	1225	4102
2040	118	2689	1421	4228
2050	124	2611	1582	4317
2060	128	2533	1721	4382
2070	130	2457	1846	4433
2080	131	2383	1963	4477
2090	130	2312	2063	4505
2100	130	2241	2159	4530

Supplementary Table 3. Change in global carbon stock and greenhouse gas emissions relative to the baseline between 2020-2100 if recovering 100% cross-laminated timber panels for power generation.

Change in carbon stock in Scenario 1 ($GtCO_2e$)									
	2020	2030	2040	2050	2060	2070	2080	2090	2100
Traditional wood products	0.0	0.1	0.3	0.6	0.9	1.2	1.3	1.2	1.0
Forest aboveground	0.0	-1.5	-3.6	-3.4	-3.9	-6.2	-7.5	-11.0	-14.6
Forest soil	0.0	0.0	-0.1	-0.2	-0.3	-0.4	-0.6	-0.7	-0.9
Forest slash	0.2	0.2	0.2	0.0	-0.2	-0.3	-0.2	-0.6	-0.6
Landfill waste	0.0	0.0	-0.1	-0.2	-0.3	-0.4	-0.6	-0.7	-0.8
Cross-laminated timber	0.0	0.0	-0.3	-0.9	-1.5	-2.1	-2.8	-3.6	-4.1
Total	0.2	-1.2	-3.5	-4.1	-5.3	-8.3	-10.4	-15.4	-20.0
Change in carbon stock in Scenario 2 ($GtCO_2e$)									
	2020	2030	2040	2050	2060	2070	2080	2090	2100
Traditional wood products	0.0	0.0	0.0	0.1	0.4	0.7	0.8	0.8	0.7
Forest aboveground	0.0	-0.6	-1.7	-3.5	-5.5	-7.5	-8.9	-12.1	-16.2
Forest soil	0.0	0.0	-0.1	-0.2	-0.3	-0.4	-0.6	-0.7	-1.0
Forest slash	0.0	0.0	0.0	0.1	0.2	0.4	0.1	-0.5	-0.5
Landfill waste	0.0	0.0	0.0	0.0	-0.1	-0.2	-0.3	-0.5	-0.6
Cross-laminated timber	0.0	0.0	0.0	-0.1	-0.5	-1.1	-1.7	-2.5	-3.2
Total	0.0	-0.6	-1.8	-3.6	-5.8	-8.2	-10.6	-15.5	-20.9
Change in carbon stock in Scenario 3 ($GtCO_2e$)									
	2020	2030	2040	2050	2060	2070	2080	2090	2100
Traditional wood products	0.0	0.2	0.6	1.2	1.8	2.3	2.7	2.9	2.9
Forest aboveground	0.0	-2.8	-6.8	-5.9	-5.4	-7.9	-8.6	-11.6	-15.6
Forest soil	0.0	0.0	-0.1	-0.3	-0.4	-0.6	-0.8	-1.0	-1.2
Forest slash	0.4	0.4	0.4	-0.2	-0.4	-0.1	0.2	-0.5	-0.9
Landfill waste	0.0	0.0	-0.1	-0.3	-0.6	-0.8	-1.1	-1.4	-1.7
Cross-laminated timber	0.0	0.0	-0.6	-1.7	-3.0	-4.3	-5.6	-7.1	-8.1
Total	0.4	-2.3	-6.6	-7.2	-8.0	-11.4	-13.3	-18.7	-24.6
Change in greenhouse gas in Scenario 1 ($GtCO_2e$)									
	2020	2030	2040	2050	2060	2070	2080	2090	2100
Biogenic carbon uptake by forest	0.2	-1.0	-3.4	-4.4	-5.8	-9.1	-11.5	-17.0	-22.9
Production and end of life of traditional wood products	0.0	-0.3	-0.9	-2.0	-3.4	-4.8	-6.3	-7.5	-8.4
Substituting market electricity	0.0	0.0	-0.2	-0.5	-0.9	-1.3	-1.7	-2.1	-2.6
Substituting traditional building materials	0.0	0.0	-0.2	-0.6	-1.1	-1.6	-2.1	-2.7	-3.3
Forest operations	0.0	0.0	0.0	0.0	0.0	0.0	0.0	0.1	0.1
Cross-laminated timber production and end of life	0.0	0.0	0.6	2.0	3.4	4.9	6.5	8.2	10.0
Recycling cross-laminated timber	0.00	0.00	0.00	0.00	0.00	0.00	0.00	0.00	0.14
Total	0.2	-1.3	-4.1	-5.6	-7.8	-11.9	-15.0	-21.1	-26.9
Change in greenhouse gas in Scenario 2 ($GtCO_2e$)									
	2020	2030	2040	2050	2060	2070	2080	2090	2100
Biogenic carbon uptake by forest	0.1	-0.5	-1.7	-3.4	-5.9	-8.7	-11.6	-17.3	-23.8
Production and end of life of traditional wood products	0.0	-0.1	-0.2	-0.5	-1.2	-2.3	-3.4	-4.4	-4.9
Substituting market electricity	0.0	0.0	0.0	-0.1	-0.3	-0.6	-1.0	-1.4	-1.9
Substituting traditional building materials	0.0	0.0	0.0	-0.1	-0.4	-0.8	-1.3	-1.9	-2.4
Forest operations	0.0	0.0	0.0	0.0	0.0	0.0	0.0	0.0	0.1
Cross-laminated timber production and end of life	0.0	0.0	0.0	0.2	1.1	2.5	4.0	5.6	7.4
Recycling cross-laminated timber	0.00	0.00	0.00	0.00	0.00	0.00	0.00	0.00	0.01
Total	0.0	-0.6	-1.8	-3.8	-6.7	-10.0	-13.4	-19.4	-25.6
Change in greenhouse gas in Scenario 3 ($GtCO_2e$)									
	2020	2030	2040	2050	2060	2070	2080	2090	2100
Biogenic carbon uptake by forest	0.5	-1.9	-6.4	-7.9	-9.2	-13.1	-15.5	-21.8	-29.3
Production and end of life of traditional wood products	0.0	-0.6	-1.8	-3.9	-6.6	-9.5	-12.5	-15.4	-18.0
Substituting market electricity	0.0	0.0	-0.3	-1.0	-1.7	-2.5	-3.3	-4.2	-5.1
Substituting traditional building materials	0.0	0.0	-0.4	-1.3	-2.2	-3.2	-4.3	-5.4	-6.5
Forest operations	0.0	0.0	0.0	0.0	0.0	0.0	0.1	0.1	0.1
Cross-laminated timber production and end of life	0.0	0.0	1.3	3.9	6.8	9.8	12.9	16.3	19.9
Recycling cross-laminated timber	0.00	0.00	0.00	0.00	0.00	0.00	0.00	0.01	0.29
Total	0.4	-2.5	-7.7	-10.2	-13.0	-18.5	-22.6	-30.3	-38.7

Supplementary Table 4. Change in global carbon stock and greenhouse gas emissions relative to the baseline between 2020-2100 if recycling 50% cross-laminated timber panels for the second use and landfilling 50% cross-laminated timber panels.

Change in carbon stock in Scenario 1 ($GtCO_2e$)									
	2020	2030	2040	2050	2060	2070	2080	2090	2100
Traditional wood products	0.0	-0.1	-0.3	-0.6	-0.9	-1.2	-1.3	-1.2	-1.0
Forest aboveground	0.0	1.5	3.6	3.4	3.9	6.2	7.5	11.0	14.6
Forest soil	0.0	0.0	0.1	0.2	0.3	0.4	0.6	0.7	0.9
Forest slash	-0.2	-0.2	-0.2	0.0	0.2	0.3	0.2	0.6	0.6
Landfill waste	0.0	0.0	0.1	0.2	0.3	0.4	0.6	0.7	1.0
Cross-laminated timber	0.0	0.0	0.3	0.9	1.5	2.1	2.8	3.6	4.1
Recycled cross-laminated timber	0.00	0.00	0.00	0.00	0.00	0.00	0.00	0.00	0.14
Total	-0.2	1.2	3.5	4.1	5.3	8.3	10.4	15.4	20.3
Change in carbon stock in Scenario 2 ($GtCO_2e$)									
	2020	2030	2040	2050	2060	2070	2080	2090	2100
Traditional wood products	0.0	0.0	0.0	-0.1	-0.4	-0.7	-0.8	-0.8	-0.7
Forest aboveground	0.0	0.6	1.7	3.5	5.5	7.5	8.9	12.1	16.2
Forest soil	0.0	0.0	0.1	0.2	0.3	0.4	0.6	0.7	1.0
Forest slash	0.0	0.0	0.0	-0.1	-0.2	-0.4	-0.1	0.5	0.5
Landfill waste	0.0	0.0	0.0	0.0	0.1	0.2	0.3	0.5	0.6
Cross-laminated timber	0.0	0.0	0.0	0.1	0.5	1.1	1.7	2.5	3.2
Recycled cross-laminated timber	0.00	0.00	0.00	0.00	0.00	0.00	0.00	0.00	0.01
Total	0.0	0.6	1.8	3.6	5.8	8.2	10.6	15.5	20.9
Change in carbon stock in Scenario 3 ($GtCO_2e$)									
	2020	2030	2040	2050	2060	2070	2080	2090	2100
Traditional wood products	0.0	-0.2	-0.6	-1.2	-1.8	-2.3	-2.7	-2.9	-2.9
Forest aboveground	0.0	2.8	6.8	5.9	5.4	7.9	8.6	11.6	15.6
Forest soil	0.0	0.0	0.1	0.3	0.4	0.6	0.8	1.0	1.2
Forest slash	-0.4	-0.4	-0.4	0.2	0.4	0.1	-0.2	0.5	0.9
Landfill waste	0.0	0.0	0.1	0.3	0.6	0.8	1.1	1.4	1.9
Cross-laminated timber	0.0	0.0	0.6	1.7	3.0	4.3	5.6	7.1	8.1
Recycled cross-laminated timber	0.00	0.00	0.00	0.00	0.00	0.00	0.00	0.01	0.28
Total	-0.4	2.3	6.6	7.2	8.0	11.4	13.3	18.7	25.2
Change in greenhouse gas in Scenario 1 ($GtCO_2e$)									
	2020	2030	2040	2050	2060	2070	2080	2090	2100
Biogenic carbon uptake by forest	0.2	-1.0	-3.4	-4.4	-5.8	-9.1	-11.5	-17.0	-22.9
Production and end of life of traditional wood products	0.0	-0.3	-0.9	-2.0	-3.4	-4.8	-6.3	-7.5	-8.4
Substituting market electricity	0.0	0.0	-0.2	-0.5	-0.9	-1.3	-1.7	-2.1	-2.6
Substituting traditional building materials	0.0	0.0	-0.2	-0.6	-1.1	-1.6	-2.1	-2.7	-3.3
Forest operations	0.0	0.0	0.0	0.0	0.0	0.0	0.0	0.1	0.1
Cross-laminated timber production and end of life	0.0	0.0	0.6	2.0	3.4	4.9	6.5	8.2	10.0
Recycling cross-laminated timber	0.00	0.00	0.00	0.00	0.00	0.00	0.00	0.00	0.01
Total	0.2	-1.3	-4.1	-5.6	-7.8	-11.9	-15.0	-21.1	-27.0
Change in greenhouse gas in Scenario 2 ($GtCO_2e$)									
	2020	2030	2040	2050	2060	2070	2080	2090	2100
Biogenic carbon uptake by forest	0.1	-0.5	-1.7	-3.4	-5.9	-8.7	-11.6	-17.3	-23.8
Production and end of life of traditional wood products	0.0	-0.1	-0.2	-0.5	-1.2	-2.3	-3.4	-4.4	-4.9
Substituting market electricity	0.0	0.0	0.0	-0.1	-0.3	-0.6	-1.0	-1.4	-1.9
Substituting traditional building materials	0.0	0.0	0.0	-0.1	-0.4	-0.8	-1.3	-1.9	-2.4
Forest operations	0.0	0.0	0.0	0.0	0.0	0.0	0.0	0.0	0.1
Cross-laminated timber production and end of life	0.0	0.0	0.0	0.2	1.1	2.5	4.0	5.6	7.4
Recycling cross-laminated timber	0.00	0.00	0.00	0.00	0.00	0.00	0.00	0.00	0.00
Total	0.0	-0.6	-1.8	-3.8	-6.7	-10.0	-13.4	-19.4	-25.6
Change in greenhouse gas in Scenario 3 ($GtCO_2e$)									
	2020	2030	2040	2050	2060	2070	2080	2090	2100
Biogenic carbon uptake by forest	0.5	-1.9	-6.4	-7.9	-9.2	-13.1	-15.5	-21.8	-29.3
Production and end of life of traditional wood products	0.0	-0.6	-1.8	-3.9	-6.6	-9.5	-12.5	-15.4	-18.0
Substituting market electricity	0.0	0.0	-0.3	-1.0	-1.7	-2.5	-3.3	-4.2	-5.1
Substituting traditional building materials	0.0	0.0	-0.4	-1.3	-2.2	-3.2	-4.3	-5.4	-6.5
Forest operations	0.0	0.0	0.0	0.0	0.0	0.0	0.1	0.1	0.1
Cross-laminated timber production and end of life	0.0	0.0	1.3	3.9	6.8	9.8	12.9	16.3	19.9
Recycling cross-laminated timber	0.00	0.00	0.00	0.00	0.00	0.00	0.00	0.00	0.02
Total	0.4	-2.5	-7.7	-10.2	-13.0	-18.5	-22.6	-30.3	-38.9

Supplementary Table 5. Summary of cumulative greenhouse gas consequences of each cross-laminated timber scenario in 2100 compared to the baseline (no cross-laminated timber).

Greenhouse gas consequences [a]	Scenario 1 Medium demand and fast adoption	Scenario 2 Medium demand and slow adoption	Scenario 3 High demand and fast adoption
Consequences to biogenic carbon uptake by wood			
Biogenic carbon uptake by wood for cross-laminated timber ($GtCO_2e$)	-14.3	-10.6	-28.6
Lost biogenic carbon uptake for decreased traditional harvested wood products ($GtCO_2e$) [a]	+7.6	+4.5	+17.0
Biogenic carbon uptake that is stored in forest ($GtCO_2e$)	-16.1	-17.7	-17.7
Net change in biogenic carbon uptake ($GtCO_2e$)	-22.9	-23.8	-29.3
Consequences to greenhouse gas emission of production and end-of-life			
Greenhouse gas emissions from cross-laminated timber production and end-of-life ($GtCO_2e$)	+10.0	+7.4	+19.9
Greenhouse gas emissions from traditional wood products production and end-of-life ($GtCO_2e$)	-8.4	-4.9	-18.0
Avoided greenhouse gas emissions from using wood waste for electricity ($GtCO_2e$)	-2.6	-1.9	-5.1
Avoided greenhouse gas emissions from substituted traditional building materials ($GtCO_2e$)	-3.3	-2.4	-6.5
Greenhouse gas emissions by forest operations (in $GtCO_2e$)	+0.1	+0.1	+0.1
Net change in greenhouse gas emissions of production and end-of-life	-4.1	-1.8	-9.6
Total net greenhouse gas emissions ($GtCO_2e$)	-27.0	-25.6	-39.0
Cross-laminated timber produced (billion m^3)	4.8	3.6	9.6
Net greenhouse gas emissions per m^3 cross-laminated timber ($tCO_2e\ m^{-3}$)	-5.6	-7.2	-4.0

[a] Positive values imply more greenhouse gases in the atmosphere (more emission to the atmosphere or less biogenic carbon uptake from the atmosphere) and negative values imply less greenhouse gases in the atmosphere (less emission to the atmosphere or more biogenic carbon uptake from the atmosphere) relative to the baseline all measured in $GtCO_2e$.

Supplementary Table 6. Notations and corresponding descriptions for the main text.

Notations	Descriptions
ρ^t	Discount factor
Q_t^{tot}	Total wood harvest
Q_t^{ind}	Wood for traditional wood production (pulp plus sawtimber)
Q_t^{CLT}	Wood for cross-laminated timber production
Z_t	Global Gross Domestic Product per capita
$D(Q_t^{ind}, Z_t)$	Global demand function for traditional wood products
C_H^i	Cost of harvesting and transporting wood to the mill
C_G^i	Cost of planting
m	Forest management intensity
G_t	Forest area regenerated planting
i	Forest type (e.g., plantation, managed, natural)
a	Forest age
t	Time
N_t^i	New forestland
$X_{a,t}^i$	Forest area type i, age a, at time t
$R_t^i\left(\sum_a X_{a,t}^i\right)$	Rental costs for land
A_t	Demand constant
θ	Income elasticity
ω	Price elasticity
$H_{a,t}^i$	Area of harvested forests
$V_{a,t}^i$	Wood yield
σ^i	Coefficient to converts biomass to carbon (species dependent)
$TFCP_t^i$	Total above ground carbon
$SOLC_t^i$	Total forest soil carbon
$\bar{K}$	Steady state level of carbon in forest soils
μ^i	Growth rate for soil carbon
AS_t^i	Carbon in forest slash (flux)
SP_t^i	Stock of carbon in slash
ϑ^i	Forest slash decomposition rate
$C\_GTM_{t,n}$	Total forest carbon stock at time *t*, in region *n*
$CLT\_C_{t,n}$	Carbon stored in cross-laminated timber
$CLT\_EOL\_C_{t,n}$	Carbon stored in cross-laminated timber end-of-life sites
$\Delta C\_GTM_{t,n}$	Forest carbon stock changes compared to the baseline
$\Delta TWP\_C_{t,n}$	Carbon stock changes in traditional wood products compared to the baseline
$Forest\_Seq_{i,t}$	Total net forest biogenic carbon flow from the atmosphere compared to the baseline
$\Delta TimberC_{t,n}^{CLT}$	Forest output carbon for cross-laminated timber
$\Delta TimberC_{t,n}^{Sawtimber}$	Forest output carbon of sawtimber
$\Delta TimberC_{t,n}^{Pulpwood}$	Forest output carbon of pulpwood
$GHG_{Forest_{t,n}}$	Life-cycle greenhouse gas emissions by forest operations
$GHG_{CLT_{t,n}}$	Life-cycle greenhouse gas emissions by producing and using cross-laminated timber
$GHG_{TWP_{t,n}}$	Life-cycle greenhouse gas emissions of producing and using traditional wood products
$GHG_{CLT\_production\_EOL_{t,n}}$	Life-cycle greenhouse gas emissions related to cross-laminated timber production and end-of-life

${GHG_{Sub\_electricity}}_{t,n}$	Potential substitution benefits of recovering the mill byproduct for power generation
${GHG_{Sub\_building}}_{t,n}$	Potential substitution benefits of replacing traditional building materials
$\Delta TWP\_C_{t,n}^{l}$	Carbon in traditional wood products converted from the wood class *l*
l	Wood class, sawtimber or pulpwood
$\Delta TWP\_In_{t,n}^{l}$	Carbon input of wood products made from timber class *l* produced in year *t* and region *n* compared to the baseline
f_m^l	Factor describing how much biogenic carbon left in wood products after manufacturing
EF^l	Manufacturing fossil greenhouse gas emission factor of producing wood products
$NewA_{i,t}$	Annual newly constructed urban commercial and residential building floor area
f_{CLT}	Cross-laminated timber usage factor per m^2 floor area
c_{CLT}	Conversion factor for cross-laminated timber from timber
r_t	Adoption rate of cross-laminated timber

Supplementary Table 7. Ecoinvent 3.9 cut-off database processes used[12].

<table>
<thead>
<tr><th>Product</th><th>Process</th></tr>
</thead>
<tbody>
<tr><td>Urea</td><td>market for urea | urea | Cutoff, U_RoW</td></tr>
<tr><td>Triple superphosphates</td><td>market for triple superphosphate | triple superphosphate | Cutoff, U_RoW</td></tr>
<tr><td>Glyphosate</td><td>market for glyphosate | glyphosate | Cutoff, U _GLO</td></tr>
<tr><td>Diesel</td><td>market for diesel | diesel | Cutoff, U_RoW</td></tr>
<tr><td>Wood chipping at forest road</td><td>market for wood chipping, chipper, mobile, diesel, at forest road | wood chipping, chipper, mobile, diesel, at forest road | Cutoff, U_GLO</td></tr>
<tr><td>Transportation</td><td>market for transport, freight, lorry >32 metric ton, EURO6 | transport, freight, lorry >32 metric ton, EURO6 | Cutoff, U_RoW</td></tr>
<tr><td>Electricity</td><td>market_group_for_electricity__high_voltage___electricity__high_voltage___Cutoff__U_Global</td></tr>
<tr><td>Natural gas</td><td>market group for natural gas, high pressure | natural gas, high pressure | Cutoff, U_GLO</td></tr>
<tr><td>Gasoline</td><td>market for petrol, unleaded | petrol, unleaded | Cutoff, U_RoW</td></tr>
<tr><td>Reinforcing steel</td><td>market for reinforcing steel | reinforcing steel | Cutoff, U_GLO</td></tr>
<tr><td>Steel</td><td>market_for_steel__low_alloyed__ steel__low_alloyed__ Cutoff__U_GLO</td></tr>
<tr><td>Concrete</td><td>market_group_for_concrete__normal_strength___concrete__normal_strength___Cutoff__U_GLO</td></tr>
<tr><td>Landfill of waste steel</td><td>treatment of scrap steel, inert material landfill | scrap steel | Cutoff, U_RoW</td></tr>
<tr><td>Sulfate pulp</td><td>sulfate pulp production, from hardwood, bleached | sulfate pulp, bleached | Cutoff, U_RoW</td></tr>
<tr><td>Sulfate pulp</td><td>sulfate pulp production, from softwood, unbleached | sulfate pulp, unbleached | Cutoff, U_RoW</td></tr>
<tr><td>Sulfite pulp</td><td>sulfite pulp production, bleached | sulfite pulp, bleached | Cutoff, U_RoW</td></tr>
<tr><td>Chemi-thermomechanical pulp</td><td>chemi-thermomechanical pulp production | chemi-thermomechanical pulp | Cutoff, U_RoW</td></tr>
<tr><td>Thermo-mechanical pulp</td><td>thermo-mechanical pulp production | thermo-mechanical pulp | Cutoff, U_RoW</td></tr>
<tr><td>Stone groundwood pulp</td><td>stone groundwood pulp production | stone groundwood pulp | Cutoff, U_RoW</td></tr>
<tr><td>Particle board</td><td>particle board production, uncoated, average glue mix | particleboard, uncoated | Cutoff, U_RoW</td></tr>
<tr><td>Particle board</td><td>particleboard production, uncoated, from virgin wood | particleboard, uncoated | Cutoff, U_RoW</td></tr>
<tr><td>Plywood</td><td>plywood production | plywood | Cutoff, U_RoW</td></tr>
<tr><td>Fibreboard</td><td>fibreboard production, hard | fibreboard, hard | Cutoff, U_RoW</td></tr>
<tr><td>Fibreboard</td><td>fibreboard production, soft, from wet & dry processes | fibreboard, soft | Cutoff, U_RoW</td></tr>
<tr><td>Sawnwood</td><td>sawnwood production, softwood, dried (u=10%), planed | sawnwood, softwood, dried (u=10%), planed | Cutoff, U_RoW</td></tr>
<tr><td>Sawnwood</td><td>sawnwood production, softwood, dried (u=20%), planed | sawnwood, softwood, dried (u=20%), planed | Cutoff, U_RoW</td></tr>
<tr><td>Sawnwood</td><td>sawnwood production, hardwood, dried (u=10%), planed | sawnwood, hardwood, dried (u=20%), planed | Cutoff, U_RoW</td></tr>
<tr><td>Sawnwood</td><td>sawnwood production, hardwood, dried (u=20%), planed | sawnwood, hardwood, dried (u=20%), planed | Cutoff, U_RoW</td></tr>
</tbody>
</table>

Supplementary Table 8. Parameters for wood, lumber, and cross-laminated timber production.

Parameter	Unit	Value
Timber production		
Wood moisture content [27]	% *dry basis*	81
Wood density[27–29]	dry kg m^{-3}	447
Wood carbon content[27,30]	% *dry basis*	50
Diesel consumption of site preparation and planting[31,32]	kg ha^{-1}	23.4
Diesel consumption of applying fertilizers and herbicides[31]	kg ha^{-1}	7.50
Diesel consumption in logging[1]	kg m^{-3}	1.66
Diesel consumption of felling[33]	kg m^{-3}	0.31
Nitrogen fertilizer usage[34]	kg N ha^{-1}	103
Phosphorus fertilizer usage[34]	kg P_2O_5 ha^{-1}	12.8
Herbicide usage (glyphosate)[31]	kg ha^{-1}	1.4
Lumber Production		
Bark mass fraction[27]	%	11
Electricity consumption of sawing[1,35–38]	kWh m^{-3} log input	24.4
Electricity consumption of kiln drying and kiln heat generation[1,2,35,36,38,39]	kWh m^{-3} lumber input	26.9
Electricity consumption of planing[1,2,35,36,38]	kWh m^{-3} lumber input	18.2
Diesel consumption of hauling materials[1,27–29]	kg m^{-3} dried lumber	3.6
Gasoline consumption of hauling materials[1,27–29]	kg m^{-3} dried lumber	0.23
Wet sawdust mass fraction of sawing byproducts[1]	%	17.9
Lumber yield rate in sawing[1,2,35,36,40–50]	%	50.0
Lumber target moisture content[1,2,5,43,44,49,51–53]	% (*dry basis*)	13.5
Overall energy efficiency for energy generation and drying[1,35,36,39]	%	17.9
Lumber drying shrinkage[1,43,44,54]	%	9.9
Planing byproduct mass percentage[1,2,35,36,55]	%	17.7
Cross-laminated Timber Production		
Resin (melamine formaldehyde) for finger-jointing and pressing[5,52,53,56]	kg/m^3 lumber input	6.1
Planing shavings percentage[52,53]	%	4.0
End cutting waste percentage[52,53]	%	12.8
Finger-jointing waste percentage[52,53]	%	0.93
Total electricity consumption of cross-laminated timber production[52,53,56,57]	kWh m^{-3} cross-laminated timber produced	113.8
Cross-laminated timber life span[58]	years	60
c_{CLT}, conversion factor for cross-laminated timber from sawtimber	m^3 cross-laminated timber per m^3 wet sawtimber	0.277

Supplementary Table 9. Parameters for landfill of wood waste.

Parameter [a]	Unit	Value
DOC_f [14,16,59,60]		0.50
MCF [14,16,59,60]		0.75
F [14,16,59,60]		0.50
OX [14,16,59,60]		0.1
k_{LF} [1,14,16,59,60]		0.01
Volume rate of CH_4 to CO_2 in landfill gas emissions[61]		1.60
Landfill gas recovery efficiency[17]	%	75
Power generation efficiency by landfill gas incineration[17]	%	30

[a] DOC_f is the degradable organic carbon fraction of wood waste that can decompose; MCF is the methane correction factor; F is the volume fraction of methane in landfill gas before recovery; OX is the average oxidation factor; k_{LF} is the landfill decay rate.

Supplementary Table 10. Average structural material usage for 1 m^2 floor area[10,11].

Material	Unit	Steel & concrete building	Cross-laminated timber building
Steel frame	kg	30.8	27.7
Concrete	kg	230	
Steel deck	kg	14.3	
Reinforcing bar	kg	8.9	
Cross-laminated timber (also referred as f_{CLT} in equation (8) in main text)	m^3		0.12

Supplementary Table 11. Parameters and values for modeling greenhouse gas emissions of traditional wood products[12,62,63].

Parameter [a]	Unit	Value
Global production share for sawtimber	%	53%
Global production share for plywood	%	20%
Global production share for particle board	%	13%
Global production share for fiber board	%	14%
Global production share for mechanical pulp	%	15%
Global production share for chemical pulp	%	85%
f_m for sawntimber		0.56
f_m for plywood		0.47
f_m for particle board		0.53
f_m for fiber board		0.44
f_m for mechanical pulp		0.96
f_m for chemical pulp		0.49
EF for sawntimber	tCO_2e per t C of sawtimber fed in	0.15
EF for plywood	tCO_2e per t C of sawtimber fed in	2.00
EF for particle board	tCO_2e per t C of sawtimber fed in	0.69
EF for fiber board	tCO_2e per t C of sawtimber fed in	0.79
EF for mechanical pulp	tCO_2e per t C of pulpwood fed in	3.78
EF for chemical pulp	tCO_2e per t C of pulpwood fed in	0.77

[a] f_m is the factor describing how much biogenic carbon left in wood products after manufacturing; *EF* is the average emission factor of fossil greenhouse gas emissions.

Supplementary Table 12. λ for each region floor area ridge regression model.

Region [a]	Urban residential floor area projection	Urban commercial floor area projection
AFME	0.0005	0.001
BRAZIL	0.0005	0.002
CANADA	0.0001	0.08
CENT AMER.	0.08	0.3
CHINA	0.1	0.0005
E ASIA	0.0001	0.0001
WEU	0.0001	0.08
EEU	0.08	0.08
JAPAN	0.0001	0
OCEANIA	0.08	0.1
RSAM	0.3	0.3
RUSSIA	0.08	0.08
SE ASIA	0.0001	0.0001
SOUTH ASIA	0.01	0.002
SSAF	0.0001	0.0001
US	0.0001	0.5

a There are 16 regions: WEU (West Europe), US (United States of America), CHINA, CANADA, SOUTH ASIA, RUSSIA, EEU (East Europe), E ASIA (East Asia), BRAZIL, JAPAN, CENT AMERICA (Central America), SSAF (Sub-Saharan Africa), OCEANIA, AFME (North Africa and Middle East), RSAM (Rest of South America), and SE ASIA (Southeast Asia).

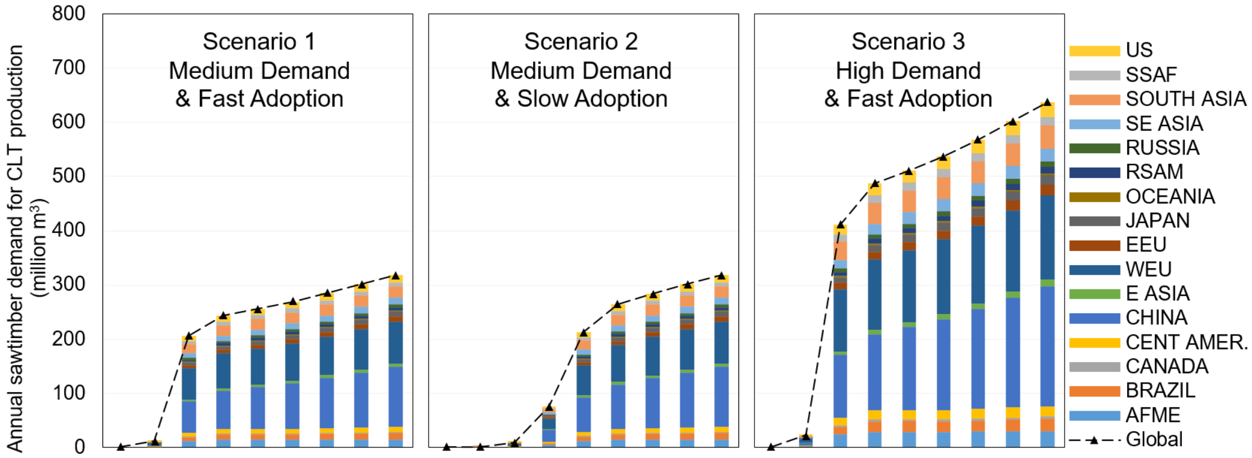

Supplementary Fig. 1. Projected demand of sawtimber for cross-laminated timber production from 2020 to 2100. There are 16 regions: WEU (West Europe), US (United States of America), CHINA, CANADA, SOUTH ASIA, RUSSIA, EEU (East Europe), E ASIA (East Asia), BRAZIL, JAPAN, CENT AMERICA (Central America), SSAF (Sub-Saharan Africa), OCEANIA, AFME (North Africa and Middle East), RSAM (Rest of South America), and SE ASIA (Southeast Asia). CLT stands for cross-laminated timber.

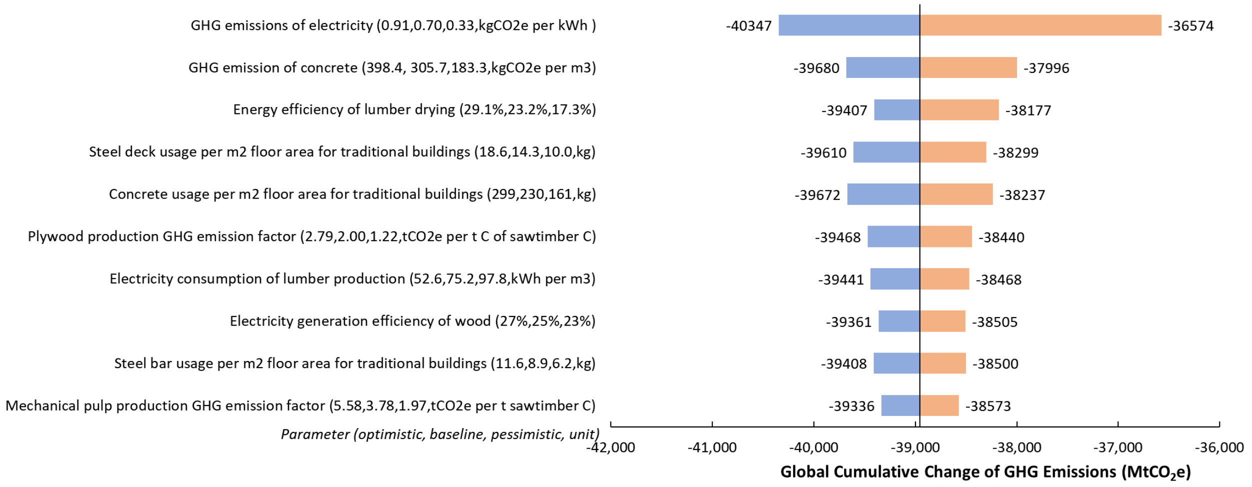

Supplementary Fig. 2. Sensitivity analysis of global cumulative change in greenhouse gas emissions relative to the baseline from 2020 to 2100 in Scenario 3. The parameters and variables with less than 1% impact on the greenhouse gas (GHG) emission results are not presented given the minor impacts.

Supplementary References

1. Lan, K., Kelley, S. S., Nepal, P. & Yao, Y. Dynamic life cycle carbon and energy analysis for cross-laminated timber in the Southeastern United States. *Environ. Res. Lett.* **15**, 124036 (2020).
2. Bergman, R. D. & Bowe, S. A. Environmental impact of manufacturing softwood lumber in northeastern and north central United States. *Wood Fiber Sci.* **42**, 67–78 (2010).
3. Bowyer, J. L., Shmulsky, R. & Haygreen, J. G. *Forest products and wood science: an introduction*. (Blackweel Publishing, 2003).
4. ANSI/APA. *ANSI/APA PRG 320-2019 Standard for Performance-Rated Cross-Laminated Timber*. (2019).
5. Gu, M. Strength and Serviceability Performances of Southern Yellow Pine Cross-Laminated Timber (CLT) and CLT-Glulam Composite Beam. (Clemson University, 2017).
6. Brandner, R., Flatscher, G., Ringhofer, A., Schickhofer, G. & Thiel, A. Cross laminated timber (CLT): overview and development. *Eur. J. Wood Wood Prod.* **74**, 331–351 (2016).
7. Hasburgh, L. *et al.* Effect of adhesives and ply configuration on the fire performance of Southern pine cross-laminated timber. in *WCTE 2016 - World Conference on Timber Engineering* (2016).
8. Wang, G. Hygrothermal Performance of Southern Pine Cross-laminated Timber. (North Carolian State University, 2018).
9. Popovski, M. & Gavric, I. Performance of a 2-Story CLT House Subjected to Lateral Loads. *J. Struct. Eng. (United States)* **142**, 1–12 (2016).
10. D'Amico, B., Pomponi, F. & Hart, J. Global potential for material substitution in building construction: The case of cross laminated timber. *J. Clean. Prod.* **279**, 123487 (2021).
11. Caruso, M. C., Menna, C., Asprone, D. & Prota, A. LCA-Based Comparison of the Environmental Impact of Different Structural Systems. *IOP Conf. Ser. Mater. Sci. Eng.* **442**, (2018).
12. Wernet, G. *et al.* The ecoinvent database version 3 (part I): overview and methodology. *Int. J. Life Cycle Assess.* **21**, 1218–1230 (2016).
13. Passarelli, R. N. The environmental impact of reused CLT panels: Study of a single-storey commercial building in Japan. *WCTE 2018 - World Conf. Timber Eng.* (2018).
14. Towprayoon, S. *et al.* Chapter 3: Solid Waste Disposal. in *2019 Refinement to the 2006 IPCC Guidelines for National Greenhouse Gas Inventories* vol. 5 6.1-6.49 (2019).
15. US EPA. Landfill Methane Outreach Program (LMOP). https://www.epa.gov/lmop/project-and-landfill-data-state (2022).
16. IPCC. Chapter 5 Waste. in *Good practice guidance and uncertainty management in national greenhouse gas inventories* (2000).
17. Anshassi, M., Sackles, H. & Townsend, T. G. A review of LCA assumptions impacting whether landfilling or incineration results in less greenhouse gas emissions. *Resour. Conserv. Recycl.* **174**, 105810 (2021).
18. Johnston, C. M. T. & Radeloff, V. C. Global mitigation potential of carbon stored in harvested wood products. *Proc. Natl. Acad. Sci. U. S. A.* **116**, 14526–14531 (2019).
19. Rüter, S., Matthews, R. W., Lundblad, M., Sato, A. & Hassan, R. A. *Volume 4: Agriculture, Forestry and Other Land Use. Ch. 12 Harvested Wood Products*. *2019 Refinement to the 2006 IPCC Guidelines for National Greenhouse Gas Inventories* (2019).
20. The World Bank. Total Population. https://data.worldbank.org/indicator/SP.POP.TOTL?view=chart (2022).

21. The World Bank. Urban population. https://data.worldbank.org/indicator/SP.URB.TOTL?year_high_desc=true (2022).
22. Deetman, S. *et al.* Modelling global material stocks and flows for residential and service sector buildings towards 2050. *J. Clean. Prod.* **245**, 118658 (2020).
23. Larasatie, P., Albee, R., Muszynski, L., Guerrero, J. M. & Hansen, E. Global CLT industry survey: The 2020 updates. in *World Conference on Timber Engineering; WCTE* 1–8 (2020).
24. Jaiswal, K. & Wald, D. J. *Creating a Global Building Inventory for Earthquake Loss Assessment and Risk Management*. (2018).
25. Rao, K. U. & Kishore, V. V. N. A review of technology diffusion models with special reference to renewable energy technologies. *Renew. Sustain. Energy Rev.* **14**, 1070–1078 (2010).
26. Purohit, P. & Kandpal, T. C. Renewable energy technologies for irrigation water pumping in India: Projected levels of dissemination, energy delivery and investment requirements using available diffusion models. *Renew. Sustain. Energy Rev.* **9**, 592–607 (2005).
27. Miles, P. D. & Smith, W. B. Specific Gravity and Other Properties of Wood and Bark for 156 Tree Species Found in North America. *Res. Note. NRS-38* 35 (2009).
28. Patterson, D. W., Doruska, P. F. & Posey, T. Weight and bulk density of loblolly pine plywood logs in southeast Arkansas. *For. Prod. J.* **54**, 145–149 (2004).
29. Patterson, H. T. & Clark, A. I. Bulk density of southern pine logs. *For. Prod. J.* **38**, 36–40 (1988).
30. Edmunds, C. W. *et al.* Blended Feedstocks for Thermochemical Conversion : Biomass Characterization and Bio-Oil Production From Switchgrass-Pine Residues Blends. *Front. Energy Res.* **6**, 1–16 (2018).
31. Markewitz, D. Fossil fuel carbon emissions from silviculture : Impacts on net carbon sequestration in forests. *For. Ecol. Manage.* **236**, 153–161 (2006).
32. Puettmann, M., Oneil, E., Milota, M. & Johnson, L. *Cradle to Gate Life Cycle Assessment of Softwood Lumber Production from the Southeast*. (2013) doi:10.13140/RG.2.2.12807.60327.
33. Oneil, E. E. *et al.* Life-Cycle Impacts of Inland Northwest and Northeast / North Central Forest Resources. *Wood Fiber Sci.* **42**, 29–51 (2010).
34. Amateis, R. L., Liu, J., Ducey, M. J. & Allen, H. L. Modeling Response to Midrotation Nitrogen and Phosphorus Fertilization in Loblolly Pine Plantations. *South. J. Appl. For.* **24**, 207–212 (2000).
35. Milota, M. R., West, C. D. & Hartley, I. D. Gate-to-gate life-cycle inventory of softwood lumber production. *Wood Fiber Sci.* **37**, 47–57 (2005).
36. Milota, M. R. *CORRIM : Phase I Final Report Module B Softwood Lumber - Pacific Northwest Region*. (2004).
37. Devaru, D. G., Maddula, R., Grushecky, S. T. & Gopalakrishnan, B. Motor-based energy consumption in west virginia sawmills. *For. Prod. J.* **64**, 33–40 (2014).
38. Milota, M. CORRIM REPORT: Module C-Life Cycle assessment for the production of southeastern softwood lumber. (2015).
39. Ananias, R. A. *et al.* Energy Consumption in Industrial Drying of Radiata Pine. *Dry. Technol.* **30**, 774–779 (2012).
40. Plank, M. E. *Lumber recovery from ponderosa pine in the Black Hills, South Dakota /.* (2014) doi:10.5962/bhl.title.94229.

41. Liu, C., Ruel, J. C., Groot, A. & Zhang, S. Y. Model development for lumber volume recovery of natural balsam fir trees in Quebec, Canada. *For. Chron.* **85**, 870–877 (2009).
42. Keegan III, C. E., Morgan, T. A., Blatner, K. A. & Daniels, J. M. Trends in Lumber Processing in the Western United States. Part II: Overrun and Lumber Recovery Factors. *For. Prod. J.* **60**, 140–149 (2010).
43. Bergman, R. D. & Bowe, S. A. Environmental impact of producing hardwood lumber using life-cycle inventory. *Wood Fiber Sci.* **40**, 448–458 (2008).
44. Bergman, R. D. & Bowe, S. A. Life-cycle inventory of manufacturing hardwood lumber in southeastern US. *Wood Fiber Sci.* **44**, 71–84 (2012).
45. Steele, P. H. *Factors determining lumber recovery in sawmilling*. (1984).
46. Kilborn, K. A. Lumber recovery studies of Alaska Sawmills, 1997 to 1999. *USDA For. Serv. - Gen. Tech. Rep. PNW* 14–15 (2002) doi:10.2737/PNW-GTR-544.
47. Mickaël, H., Michaël, A., Fabrice, B., Pierre, M. & Thibaud, D. Soil detritivore macro-invertebrate assemblages throughout a managed beech rotation. *Ann. For. Sci.* **64**, 219–228 (2007).
48. Lowell, E. C. & Green, D. W. Lumber Recovery From Small-Diameter Ponderosa Pine From Flagstaff, Arizona. in *USDA Forest Service Proceedings RMRS-P-22* 161–166 (2001).
49. Zhang, S., Chauret, G. & Tong, Q. Impact of precommercial thinning on tree growth, lumber recovery and lumber quality in Abies balsamea. *Scand. J. For. Res.* **24**, 425–433 (2009).
50. Wagner, F. & Taylor, F. Low lumber recovery at southern pine sawmills may be due to misshapen sawlogs. *For. Prod. J.* **43**, 53–55 (1993).
51. Bergman, R. Chapter 13 - Drying and Control of Moisture Content and Dimensional Changes. in *Wood Handbook - Wood as an engineering material* 1–20 (2010).
52. Puettmann, M., Sinha, A. & Ganguly, I. *CORRIM Report - Life cyle assessment of cross laminated timber produced in Oregon*. https://corrim.org/wp-content/uploads/2019/02/Life-Cycle-Assessment-of-Oregon-Cross-Laminated-Timber.pdf (2018).
53. Chen, C. X., Pierobon, F. & Ganguly, I. Life Cycle Assessment (LCA) of Cross-Laminated Timber (CLT) produced in Western Washington: The role of logistics and wood species mix. *Sustainability* **11**, (2019).
54. Wang, E., Chen, T., Pang, S. & Karalus, A. Variation in anisotropic shrinkage of plantation-grown pinus radiata wood. *Maderas Cienc. y Tecnol.* **10**, 243–250 (2008).
55. Blatner, K. A., Keegan, C. E., Daniels, J. M. & Morgan, T. A. Trends in lumber processing in the western united states. part III: Residue recovered versus lumber produced. *For. Prod. J.* **62**, 429–433 (2012).
56. Athena Sustainable Materials Institute. *A Life Cycle Assessment of Cross-Laminated Timber Produced in Canada*. http://www.athenasmi.org/resources/publications/ (2013).
57. Bédard, P. *et al.* Manufacturing Cross-Laminated Timber (CLT) Technological and Economic Analysis. at https://library.fpinnovations.ca/en/permalink/fpipub39323 (2010).
58. Gu, H. & Bergman, R. Life cycle assessment and environmental building declaration for the design building at the University of Massachusetts. *Gen. Tech. Rep. FPL-GTR-255. Madison, WI US Dep. Agric. For. Serv. For. Prod. Lab. 1-73.* **255**, 1–73 (2018).

59. Jensen, J. E. F. & Pipatti, R. CH_4 Emissions from Solid Waste Disposal. in *Good Practice Guidance and Uncertainty Management in National Greenhouse Gas Inventories* 339–348 (2006).
60. Pipatti, R. *et al.* Waste generation and compositon. in *2006 IPCC Guidelines for National Greenhouse Gas Inventories* (2006).
61. Chai, X. *et al.* Characteristics of environmental factors and their effects on CH_4 and CO_2 emissions from a closed landfill: An ecological case study of Shanghai. *Waste Manag.* **30**, 446–451 (2010).
62. Sun, M., Wang, Y., Shi, L. & Klemeš, J. J. Uncovering energy use, carbon emissions and environmental burdens of pulp and paper industry: A systematic review and meta-analysis. *Renew. Sustain. Energy Rev.* **92**, 823–833 (2018).
63. Tomberlin, K. E., Venditti, R. & Yao, Y. Life cycle carbon footprint analysis of pulp and paper grades in the United States using production-line-based data and integration. *BioResources* **15**, 3899–3914 (2020).
