# Supplementary material for: Global land and carbon consequences of mass timber products
Source: Nat Commun. 2025 May 26;16:4864. doi: 10.1038/s41467-025-60245-y (PMC12106697; doi:10.1038/s41467-025-60245-y)
Supplement: Supplementary file 2 — Description of Additional Supplementary File [file 41467_2025_60245_MOESM2_ESM.pdf]

Description of additional supplementary file

Supplementary Data 1 - Supplementary Data contains the supplementary data related to Global Timber Model regions, cross-laminated timber demand projection and adoption, and baseline forest area results.
